# Correlation of miRNA expression with intensity of neuropathic pain in man

**DOI:** 10.1177/1744806919860323

**Published:** 2019-07-10

**Authors:** Diana Tavares-Ferreira, Nathan Lawless, Emma V Bird, Simon Atkins, David Collier, Emanuele Sher, Karim Malki, Daniel W Lambert, Fiona M Boissonade

**Affiliations:** 1 School of Clinical Dentistry, University of Sheffield, UK; 2 Lilly Research Centre, Eli Lilly and Company, Surrey, UK

**Keywords:** miRNA, pain, behaviour, neuroma, nerve injury, lingual nerve, bioinformatics, rat, human

## Abstract

**Background:**

Peripheral nerve injury causes changes in expression of multiple receptors and mediators that participate in pain processing. We investigated the expression of microRNAs (miRNAs) – a class of post-transcriptional regulators involved in many physiological and pathophysiological processes – and their potential role in the development or maintenance of chronic neuropathic pain following lingual nerve injury in human and rat.

**Methods:**

We profiled miRNA expression in Sprague-Dawley rat and human lingual nerve neuromas using TaqMan® low-density array cards. Expression of miRNAs of interest was validated via specific probes and correlated with nerve injury-related behavioural change in rat (time spent drinking) and clinical pain (visual analogue scale (VAS) score). Target prediction was performed using publicly available algorithms; gene enrichment and pathway analysis were conducted with MetaCore. Networks of miRNAs and putative target genes were created with Cytoscape; interaction of miRNAs and target genomes in rat and human was displayed graphically using CircosPlot.

**Results:**

rno-miR-138 was upregulated in lingual nerve of injured rats versus sham controls. rno-miR-138 and rno-miR-667 expression correlated with behavioural change at day 3 post-injury (with negative (rno-miR-138) and positive (rno-miR-667) correlations between expression and time spent drinking). In human, hsa-miR-29a was downregulated in lingual nerve neuromas of patients with higher pain VAS scores (painful group) versus patients with lower pain VAS scores (non-painful). A statistically significant negative correlation was observed between expression of both hsa-miR-29a and hsa-miR-500a, and pain VAS score.

**Conclusions:**

Our results show that following lingual nerve injury, there are highly significant correlations between abundance of specific miRNAs, altered behaviour and pain scores. This study provides the first demonstration of correlations between human miRNA levels and VAS scores for neuropathic pain and suggests a potential contribution of specific miRNAs to the development of chronic pain following lingual nerve injury. Putative targets for candidate miRNAs include genes related to interleukin and chemokine receptors and potassium channels.

## Introduction 

Pain perception forms part of a protective mechanism against tissue damage, but if it evolves to a chronic state, it may no longer serve a useful biological function and can become severely disabling with significant impact on quality of life.^1^ Neuropathic pain is defined as a type of chronic pain that results from direct damage to the peripheral or central nervous system (CNS).^2^ The precise mechanisms behind the development and maintenance of chronic pain after a peripheral nerve injury are not fully understood. However, it is clearly established that nerve injury induces extensive complex changes at the injury site including recruitment of immune cells, induction and release of multiple inflammatory mediators and increased expression of a wide range of modulators of neuronal excitability including neuropeptides and voltage-gated ion channels.^3–5^ These events contribute to the sensitisation of sensory neurons, the development of altered excitability and the generation of spontaneous ectopic neuronal firing at the injury site.^4,6,7^ Disorganisation of regenerating axons and development of connective and scar tissue at the site of injury may lead to the formation of a neuroma, where spontaneous ectopic activity may continue to be generated for many years after the initial injury. This activity produces spontaneous pain, a distinct characteristic of neuropathic pain,^5^ and in addition is believed to drive central change, further enhancing the chronicity of the pain.

Significant evidence now exists that microRNAs (miRNAs) regulate a multiplicity of cellular processes, including immune and inflammatory responses.^6,8^ miRNAs are small noncoding RNAs that regulate the expression of multiple genes, predominantly by interacting with the 3′ untranslated region of messenger RNAs (mRNAs).^7^ Neuropathic pain is characterised by alteration in the expression of receptors and mediators (as described above), and there is evidence that miRNAs may contribute to some of these changes.^9,10^ For instance, miR-96^11^ and miR-7a^12^ have been reported to regulate the sodium channel Nav1.3, and the specific deletion of Dicer (an enzyme required for the processing of pre-miRNA) in dorsal root ganglia resulted in changes in the expression of sodium channels Nav1.7, 1.8 and 1.9.^13^ Recent studies have demonstrated that different miRNAs are expressed in different pain conditions and that their expression changes over time. Tam Tam et al.^14^ demonstrated that miR-143 expression is decreased in dorsal root ganglion (DRG) in inflammatory pain caused by injection of complete Freund’s adjuvant (CFA), but not after transection of the sciatic nerve. These studies indicate that as key regulators that interact with several genes, miRNAs may be promising targets for the treatment of neuropathic pain.^15^

In this study, we have focused on the potential role of altered miRNA expression in the development of pain following lingual nerve injury, which can occur during routine oral surgery or facial trauma. Patients with this type of injury often report sensory alterations in the ipsilateral side of their tongue including loss of taste (due to the damage of gustatory afferents), hypoaesthesia (decreased sensitivity to stimulation), paraesthesia (abnormal sensations including tingling, itching) and dysaesthesia (abnormal unpleasant sensations such as burning and pain).^16^ If the symptoms are persistent, patients are referred for surgical repair of the injured nerve. Our centre provides a service for treatment of patients with these injuries, including repair of the lingual nerve, which has resulted in the collection of an extensive archive of lingual nerve neuromas and their associated clinical pain histories. Even though the overall outcome of the surgical repair is positive, when symptoms of dysaesthesia are present prior to repair, they usually persist following surgery,^17^ and the pharmacological treatment of dysaesthesia (e.g. with gabapentin or pregabalin) has limited success.^16^ In summary, to date, the sequelae of nerve injury are not fully understood and have not been therapeutically met^18–20^; thus, further investigation of the underlying mechanisms of neuropathic pain and the identification of novel potential therapeutic targets are required.

The aim of this study was to investigate the expression of miRNAs in both human lingual nerve neuromas and rat lingual nerve and to examine whether any correlation exists between the expression of miRNAs and clinical pain symptoms in patients or behavioural change in animals. In this study, we have quantified miRNA expression in human lingual nerve neuromas and rat lingual nerves (following chronic constriction injury (CCI) or sham injury) and correlated expression with clinical pain and behavioural change. Subsequently, we applied bioinformatics tools to predict potential target genes and pathways under regulatory control of the miRNAs identified. To our knowledge, this is the first study to demonstrate that expression levels of specific miRNAs in human neuromas correlate with clinical pain history and intensity.

## Methods

### Study design

A total of 18 adult male Sprague-Dawley rats (Charles River, Margate, UK; 250–300 g) were used. All animals were assessed for feeding behaviour (Ugo Basile Orofacial Stimulation Test) on day 0 (baseline (BL), before surgery) and on day 3 (after surgery; CCI or sham procedure). We have previously established that at this time point, there are high levels of spontaneous neuronal activity (implicated in the development of neuropathic pain^21^) and increased neuropeptide and sodium channel expression at the injury site.^22–32^ Lingual nerve samples were obtained from the injury site, or equivalent site in sham-operated animals (CCI *n* = 3, sham procedure *n* = 3), and processed for miRNA screening using TaqMan® low-density array (TLDA) cards. Specific miRNAs were selected and validated with reverse transcription quantitative real-time polymerase chain reaction (reverse transcription qPCR; RT-qPCR) in the same six samples.

Seventeen human lingual nerve neuromas were obtained from patients with lingual nerve injury undergoing treatment at the Charles Clifford Dental Hospital, Sheffield, UK. These were divided into ‘painful’ (*n* = 8) and ‘non-painful’ (*n* = 9) based on the pain visual analogue scale (VAS) score assessment conducted by the clinician prior to nerve repair surgery. TLDA miRNA screening was carried out on 12 human neuroma samples (painful *n* = 6, non-painful *n* = 6) to identify miRNAs linked to pain symptoms. Our previous miRNA studies using primary cells^33^ indicated that this should be achievable with this number of samples. Specific miRNAs were selected and validated with RT-qPCR in painful human neuromas (*n* = 8, six of those used for TLDA screening with two additional samples) and non-painful neuromas (*n* = 8, five of those used for TLDA screening with three additional samples).

Further bioinformatics analyses were performed to predict potential target genes for the miRNAs of interest.

### Animal experiments

A total of 18 adult male Sprague-Dawley rats (250–300 g) were acquired from Charles River, UK, and kept in a 12-h light–dark cycle with free access to water and food. All experiments were carried out in accordance with the UK Home Office Animals (Scientific Procedures) Act, 1986. The ARRIVE guidelines^34^ have been followed in reporting this study. The rat model of neuropathic pain was adapted from Bennett and Xie.^35^ Under general anaesthesia (isoflurane; 4% induction, 2%–3% maintenance), the left lingual nerve was exposed and constricted with two loosely tied 6/0 chromic catgut sutures (CCI group; *n* = 9). In the sham group, the lingual nerve was exposed but not injured (*n* = 9). The subcutaneous tissue and overlying skin was closed with 4/0 vicryl sutures (Ethicon, Norderstedt, Germany). Animals were randomly assigned to CCI or sham group and left to recover for a period of three days following surgery. The feeding behaviour of these animals was investigated before surgery and three days post-surgery (see section below). At the end of the recovery period, the animals were deeply anaesthetised with an overdose of pentobarbitone and transcardially perfused with cold phosphate-buffered saline to remove any possible blood-derived RNA/miRNA cross-contamination. Tissues were collected quickly, snap frozen in liquid nitrogen and subsequently stored at –80°C until required for further processing. In all cases, the time between animal sacrifice and tissue snap freezing was less than 10 min.

### Behavioural testing

The effect of lingual nerve injury on feeding behaviour was measured using the Orofacial Stimulation Test (code 31300, Ugo Basile, Comerio, VA, Italy).^36^ Briefly, the animals (*n* = 18) voluntarily inserted the snout through an opening and used the tongue (which is innervated by the lingual nerve) to access a reward (chocolate milk) from a bottle. The equipment automatically measured the duration of feeding over a given period of time (‘time spent drinking’), independently of investigator bias. One week prior to the surgery, animals were trained to use the behavioural equipment. Sessions consisted of a 10-min acclimatisation period plus a further 10-min recording with access to the reward bottle. Behavioural assessment took place immediately before the surgery (day 0) and on day 3 after surgery. In total, 18 animals were used for behavioural testing, but only 6 animals (CCI *n* = 3; sham *n* = 3) were used for the purpose of the miRNA study reported here.

### Human lingual nerve neuroma samples

Neuromas used in this study were obtained from the archive of lingual nerve neuromas collected from the Charles Clifford Dental Hospital, Sheffield, UK. All neuromas were collected with the written informed consent of the patients. The study was conducted in accordance with ethical approvals received from the South Sheffield Research Ethics Committee (06/Q2305/151 STH13926) and the East of England – Cambridge Central Research Ethics Committee (17/EE/0238 STH 19847). The pain history and symptoms (pain, tingling, or discomfort on the side of the tongue innervated by the injured nerve) were obtained by the clinician preoperatively. In addition, patients were also asked to score the discomfort, tingling and pain using VAS score. A detailed description of the surgical removal of the neuroma, repair of the nerve and collection of clinical history has been described previously.^17^ Based on the pain VAS scores, the neuromas chosen for this study were divided in two groups: ‘painful’ (pain VAS score higher than 30) and ‘non-painful’ (pain VAS score lower than 8). Human tissue preparation was performed following previously published protocols by Bird et al.^37^ After surgical removal, the neuromas were placed in 2% Zamboni’s fixative (0.1 mol/L phosphate buffer, pH 7.4, containing 4% paraformaldehyde and 0.2% picric acid) for 24 h at 4°C, subsequently cryoprotected in 30% sucrose solution for 12 h at 4°C and stored at –80°C.

### miRNA extraction and TLDA cards

Rat lingual nerve tissue was disrupted and miRNA isolated using the mirVana miRNA Isolation Kit (ThermoFisher, Paisley, UK) according to the manufacturer’s protocol. Human neuroma tissue miRNA was isolated using the RecoverAll Total Nucleic Acid Isolation Kit (ThermoFisher). As the samples used were frozen and not embedded in paraffin, the deparaffinisation step was omitted from the manufacturer’s protocol. Instead, prior to extraction, human tissue samples were stored in RNAlater-ICE Frozen Tissue Transition Solution (ThermoFisher) overnight at –20°C, to facilitate the thawing of the tissue while preserving RNA quality.

miRNA expression profiles were initially screened using TLDA Rodent miRNA Cards v.3 A and B, and TLDA Human miRNA Cards v.3 A and B, respectively, for rat and human lingual nerve samples. Each card held up to 381 preloaded miRNA probes and endogenous controls (according to the miRBase database,^38^ there are currently 496 verified rat miRNA sequences and 1,917 verified human miRNA sequences). All samples were reverse-transcribed using the TaqMan miRNA Reverse Transcription kit (ThermoFisher) and preamplified (12 cycles) using TaqMan PreAmp master mix and primers, prior to being loaded into the respective TLDA microfluidic cards following the manufacturer’s protocol.

### miRNA validation

The expression of miRNAs of interest selected in the TLDA screening was validated using TaqMan miRNA Assays (triplicate per sample) and performed in a Rotor Gene real-time PCR machine (Qiagen, Manchester, UK). After reverse transcription and preamplification with specific TaqMan probes for the miRNAs of interest and endogenous control (snRNAU6), Rotor-Gene 2.1.0.9 software (Qiagen) was used to set the qPCR reaction (initial enzyme activation at 95°C for 10 min, and cycling conditions defined at 95°C for 15 s followed by 60°C for 60 s, for 40 cycles). A no-template control (no PreAmp product) sample was added to test for cDNA contamination.

### Statistical analyses

#### Rat behavioural testing

The results of the Orofacial Stimulation Test (time spent drinking) were evaluated statistically: a paired *t* test (two-tailed, equal variances assumed) was used to evaluate differences within the CCI and sham groups, comparing results at day 3 with the BL; differences between groups at BL (before surgery) were evaluated using an unpaired *t* test (two-tailed, equal variances assumed). In addition, Pearson’s correlations were applied to investigate the relation between miRNA expression and time spent drinking.

#### Patient clinical variables

Differences in VAS scores between painful and non-painful groups were evaluated using an unpaired *t* test with equal variance assumed. Pearson’s correlation was used to test the association between pain VAS score and discomfort VAS score, and between miRNA expression and pain, tingling and discomfort VAS scores.

#### Analysis of TaqMan miRNA array data and miRNA validation PCR

For both human and rat TLDA array cards, the delta C_t_ normalisation method was applied and the snRNAU6 endogenous control used. Prior to normalisation, any miRNA not detected in two of either the experimental or control samples was removed from further analysis. The unpaired *t* test was performed to calculate statistical differences between groups.

For qPCR validation of miRNAs of interest selected during TLDA screening, the comparative delta-delta C_t_ (2(–ΔΔC_t_)) method was used. First, delta C_t_ (ΔC_t_) values for each miRNA were obtained after normalising to snRNAU6. The relative expression for each miRNA in the experimental lingual nerve (CCI in the rat tissue and painful group in the human tissues) was compared with that in the control lingual nerves (sham in the rat tissues and non-painful in the human tissues) and calculated using the expression 2^−ΔΔCt^, where ΔΔC_t_ = ΔC_t_(experimental) – ΔC_t_ (control). Results were considered statistically significant with *p* < 0.05.

### Target prediction and enrichment analysis

Target genes potentially regulated by miRNAs of interest were predicted using the consensus of three publicly available miRNA target databases: TargetScan (http://www.targetscan.org),^39^ MicroCosm (http://www.ebi.ac.uk/enright-srv/microcosm),^40^ and miRTarBase (http://mirtarbase.mbc.nctu.edu.tw).^41^ The predicted target false-positive rate was reduced significantly by applying a cut-off of –0.4 in the context++ scores for TargetScan results^42^; each miRNA target was cross-referenced against this gene set. Enrichment and pathway analysis of predicted miRNA gene targets was undertaken using MetaCore™ process networks, pathway maps and GO molecular functions/processes. Top pathways were ranked based on *z*-score and the additional enriched gene count per pathway. Interactions between miRNAs and their target gene networks for each tissue were visualised using CyTargetLinker v3.0.1, an open source software package for Cytoscape v.4.0.^43^ Circos v0.67 was used to display interaction between miRNAs and target genes in a circular layout, facilitating the visualisation of the position of the miRNAs and target genes in the respective genome^44^; only target genes passing the described cut-off were visualised in the CircosPlot.

## Results

### Rat lingual nerve tissues

#### Behavioural testing

**Figure 1.**
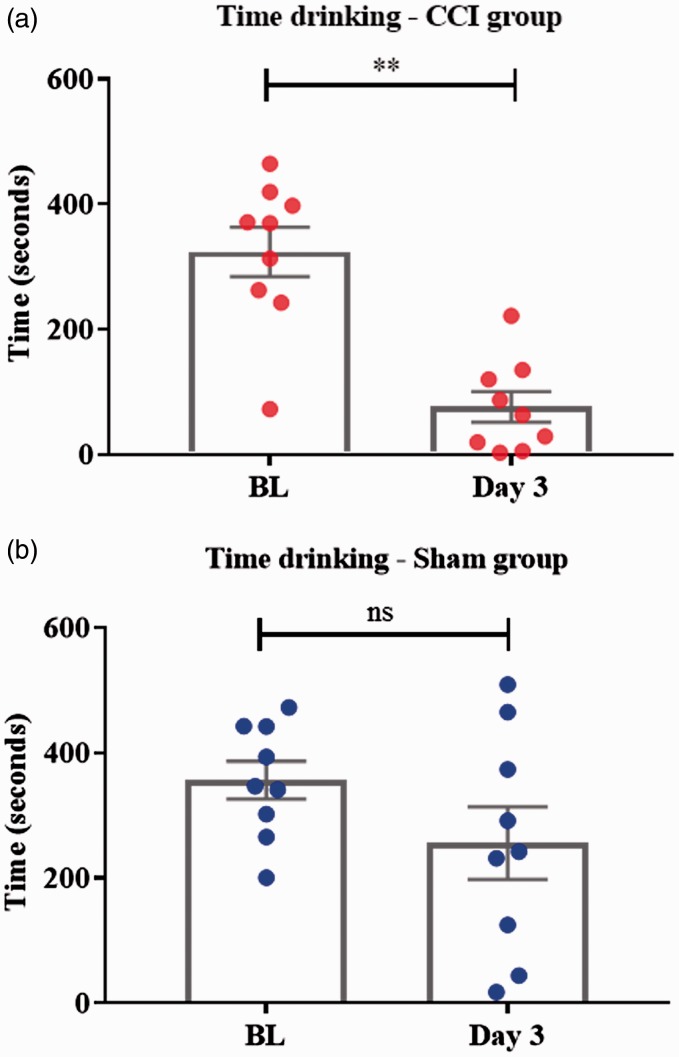
Box plot showing total time spent drinking within each group, comparing three days after injury/sham with BL. (a) In the CCI group, time spent drinking was significantly reduced on day 3 compared with BL (**p = 0.001; paired *t* test). (b) In the sham group, no statistical differences in the time spent drinking were found between BL and day 3 (ns; paired *t* test). CCI: chronic constriction injury; BL: baseline; ns: not significant.

Evaluation of the effect of surgery on the feeding behaviour (time spent drinking) within each group showed a significant decrease following injury in the experimental CCI group on day 3 compared with BL (*t* = 5.003, *df* = 8, *p* = 0.001; paired *t* test) (Figure 1(a)). In the sham group, no statistical differences were found on day 3 compared with BL (*t* = 2.2025, *df* = 8, *p* = 0.0775; paired *t* test) (Figure 1(b)). There were no statistical differences in BL values between groups (*t* = 0.658, *df* = 16, *p* = 0.5199; unpaired *t* test).

### miRNA expression in rat lingual nerve

We identified five miRNAs differentially expressed in the rat lingual nerve (Table 1). These five miRNAs sequences from the rodent TLDA cards were selected based on higher ΔΔC_t_ values and lower *p* value (*p* < 0.05). Two of these miRNAs were selected for miRNA validation: rno-miR-138 (upregulated in the CCI group vs. sham) and rno-miR-667 (downregulated in the CCI group). As TLDA cards for rodent miRNA analysis include probes for both rat and mouse, these two miRNAs were selected because they were confirmed in the miRBase database as being present in the rat genome.

**Table 1.** miRNAs differentially expressed in the rat lingual nerve.

miRNA	ΔΔC_t_	*p* value
rno-miR-667	8.55	0.03
rno-miR-138	–9.12	0.0002
mmu-miR-1957	–10.04	0.05
mmu-miR-1904	8.50	0.0002
mmu-miR-1951	12.22	0.005

Rat miRNA TLDA samples were analysed using the unpaired *t* test.

miRNA: microRNA.

**Figure 2.**
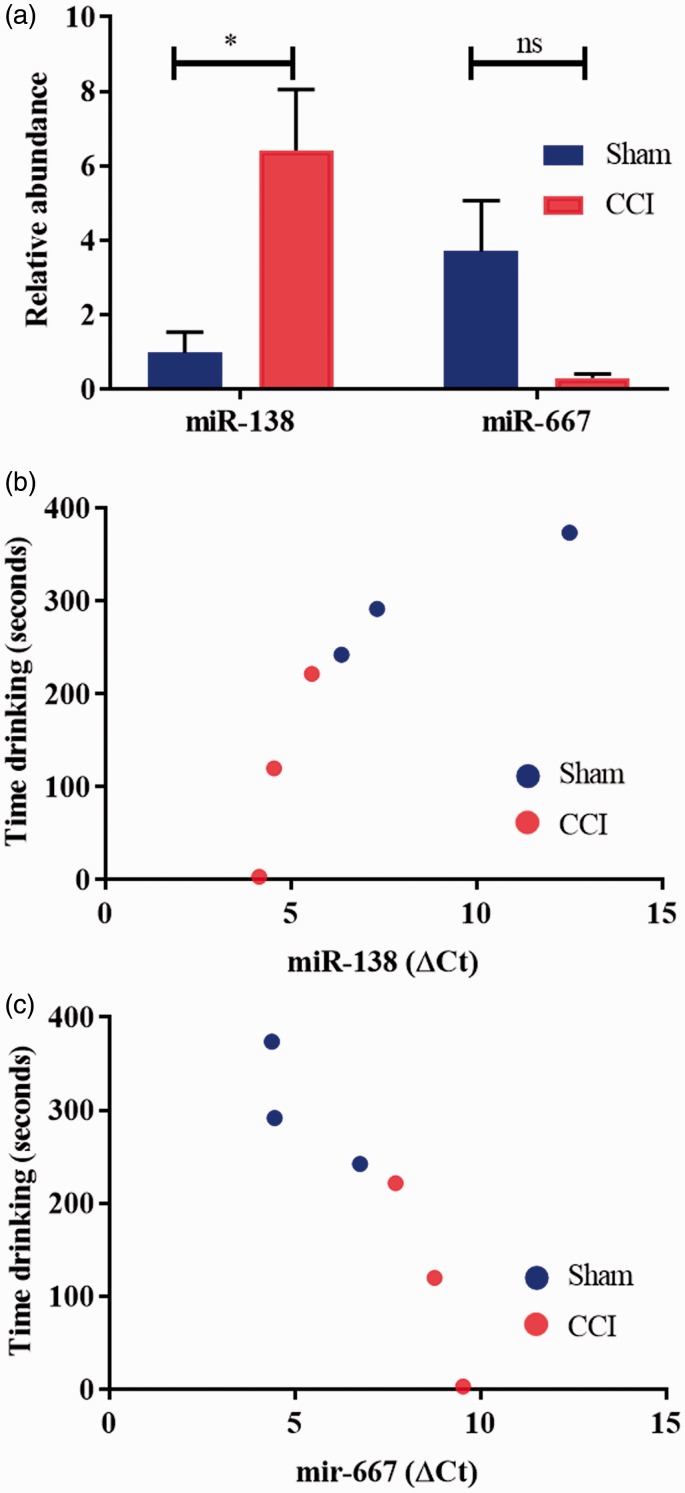
miRNA validation and correlation scatter plots. (a) miR-138 was confirmed to be statistically significantly differentially expressed between sham and CCI group (increased in the CCI group vs. sham; *p < 0.05; unpaired *t* test). miR-667 was not statistically differentially expressed in the miRNA validation study (ns; unpaired *t* test). (b) Pearson’s correlations showed a strong correlation between the expression of miR-138 and the time spent drinking on day 3 (*r* = 0.8511, *p* = 0.0316). (c) Pearson’s correlations showed a strong correlation between the expression of miR-667 and the time spent drinking on day 3 (*r* = –0.9327, *p* = 0.0066). In (b) and (c), miRNA values are provided in terms of normalised delta C_t_ values (ΔC_t_); the higher the Ct value, the lower the expression of the miRNA; thus, the expression of miR-138 is strongly negatively correlated, and the expression of miR-667 is strongly positively correlated with the time spent drinking on day 3. CCI: chronic constriction injury; ns: not significant.

The same rat lingual nerve samples were used for miRNA validation. In the validation study, only miR-138 expression was found to be significantly different (increased in the CCI vs. the sham group) at day 3 post-injury (miR-138: *t* = 3.125, *df* = 4, *p* = 0.0353, unpaired *t* test; miR-667: *t* = 2.501, *df* = 4, *p* = 0.0667, unpaired *t* test) (Figure 2(a)). However, a significant correlation was found between the normalised delta C_t_ and the time spent drinking for both miR-138 (positive correlation *r* = 0.8511, *p* = 0.0316, reflective of a negative correlation between abundance of miR-138 and time spent drinking) and miR-667 (negative correlation *r* = −0.9327, *p* = 0.0066, reflective of a positive correlation between abundance of miR-667 and time spent drinking) (Figure 2(b, c)).

### miRNA target prediction and gene enrichment analysis

**Figure 3.**
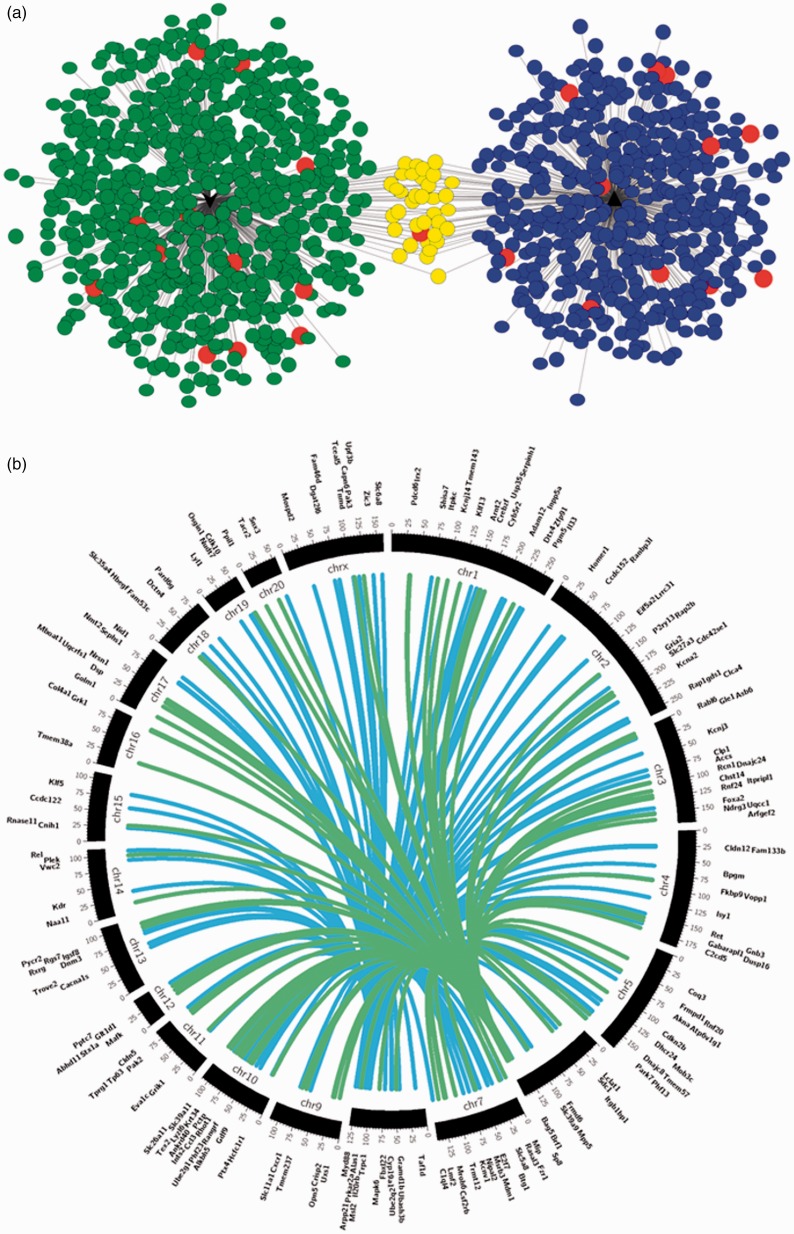
Rat miRNAs of interest and respective putative target genes. (a) CyTargetLinker analysis and network visualisation of differentially expressed rat miRNAs and their predicted target genes. Network enrichment genes for rno-miR-138 and rno-miR-667 were identified using MicroCosm, TargetScanHuman and miRTarBase. Nodes coloured in blue are targets of rno-miR-138, and nodes coloured in green are targets of rno-miR-667. The nodes coloured in yellow are targets of both rno-miR-138 and rno-miR-667, while nodes in red are genes known to have a role in pain. This network was constructed and visualised using CyTargetLinker v3.0.1 and Cytoscape v3.4.0, and there is no relation between the position of the nodes and the score of the target prediction. (b) Circos v0.67 plot of rno-miR-138 (blue) and rno-miR-667 (green) and regulatory target genes. The middle track represents the rat genome with each ideogram segment representing one chromosome. The outermost and inner tracks display the gene name and spatial location of each gene targeted by both rat miRNAs.

We next sought to identify potential functional consequences of altered expression of the candidate miRNAs. Given the correlation results obtained, we conducted target prediction and gene enrichment analyses for both rno-miR-138 and rno-miR-667. The first step was to use publicly available algorithms to look for predicted target genes of the identified miRNAs. We used TargetScan, MicroCosm and miRTarBase included in the CyTargetLinker app to build a miRNA-target gene network (Figure 3(a)). The target genes chosen for further analysis were ranked by the cumulative weighted context++ scores of TargetScan applying a cut-off of –0.4,^39,42^ resulting in 119 target genes for rno-miR-138 and 91 for rno-miR-667. rno-miR-138 is located on chromosome 8 of the rat genome and rno-miR-667 is located on chromosome 6, and the target genes are located on different chromosomes across the rat genome (Figure 3(b)). Gene enrichment and pathway analysis were carried out using MetaCore™ process networks, pathway maps, GO molecular functions and GO biological processes.

Examples of genes targeted by the miRNAs and enriched for these pathways are listed in Table S1 (please see supplementary files). The MetaCore Process networks identified seven main networks. These included neurophysiological process transmission of nerve impulse, Wnt signal transduction, cell adhesion, chemotaxis, calcium transport, inflammation and neutrophil activation and neuropeptide signalling pathways. The MetaCore pathway maps were enriched for cell adhesion and chemokines. MetaCore diseases and biomarkers enrichment analysis detected epilepsy and mouth diseases. The enrichment analysis for GO biological process included regulation of transmembrane transport, regulation of ion transport, interleukin (IL)-8-mediated signalling pathway and T cell chemotaxis. The GO molecular function identified ion transmembrane transporter activity, voltage-gated cation channel activity, potassium channel activity and ion channel binding.

### Potential novel rat miRNAs

The rodent TLDA cards are designed to specifically detect 373 rat and 641 mouse miRNAs. In the rat lingual nerve samples analysed, eight miRNA sequences were detected that have only been reported in the mouse (as in accordance with the miRBase database). However, a sequence blast against the rat genome detected a high similarity for the stem-loop precursor and mature sequences of two of these miRNA sequences, miR-1904 and miR-763; one of these (miR-1904) was found to be differentially expressed between CCI and sham groups (tool available at https://blast.ncbi.nlm.nih.gov/Blast.cgi). The stem-loop sequence of miR-1904 is located on chromosome 13 of the mouse genome, and it was identified as a high similarity sequence for the rat genome on chromosome 2 with only two mismatches (*p* = 1e-27, ident: 97%). The mature sequence (22 nucleotides) corresponds to nucleotide 11 to 32 of the stem-loop and presents a mismatch outside the seed region on the position 12 (*p* = 0.18, ident: 95%).

The miR-763 stem-loop sequence is located on chromosome 10 of the mouse genome. A high similarity on chromosome 7 on the rat genome with 3 mismatches (*p* = 5e-48, ident: 97%) was found. The mature miRNA sequence comprises nucleotides 38 to 59 and has an exact match in the rat genome (*p* = 8e-04, ident: 100%).

It is possible that these represent novel rat miRNAs; however, further experimentation required to provide validation of this (as per miRBase criteria) is beyond the scope of this study.

### Human lingual nerve neuromas

#### Analysis of clinical characteristics

**Figure 4.**
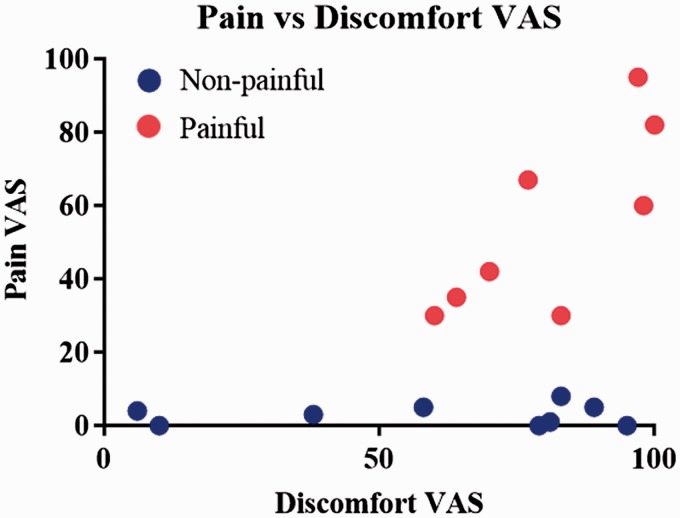
Correlation between pain and discomfort VAS scores. Pearson’s correlations showed a significant positive correlation between pain and discomfort VAS scores (*r* = 0.5, *p* = 0.05). VAS: visual analogue scale.

Clinical variables were explored to test difference in VAS scores between patients. Patients in the non-painful group reported an average pain VAS score of 2.89, which is generally considered as no pain.^45^ Patients in the painful group had VAS scores ranging between 30 and 95, with an average of 55.13. Overall, there was a significant difference in the pain VAS score between the high and low pain groups (*t* = 6.29, *df* = 15, *p* < 0.0001). Pain VAS score was found to be significantly correlated with VAS scores on discomfort (Pearson’s *r* = 0.5, *p* = 0.05) (Figure 4). No relationship was found between tingling and pain, nor between tingling and discomfort (Table 2).

**Table 2.** Clinical characteristics of the patients included in the study.

Neuroma sample	Age (years)	Sex	Time after injury (months)	Pain VAS	Tingling VAS	Discomfort VAS
Non-painful						
1^a^	42	F	20	0	33	10
2	36	F	24	0	25	79
3	27	F	5	1	1	81
4	38	F	10	3	10	38
5	60	M	–	4	7	6
6	59	F	8	5	38	58
7^b^	31	F	10	0	8	95
8^b^	47	F	8	5	94	89
9^b^	44	F	12	8	6	83
Painful						
10	35	F	6	30	6	60
11	38	M	48	30	97	83
12	30	F	12	35	75	64
13	54	F	36	60	11	98
14	44	F	7	82	85	100
15	44	F	9	95	3	97
16^b^	29	F	12	42	0	70
17^b^	47	F	10	67	100	77

Data missing for time after injury for neuroma sample 5.

F: female; M: male; VAS: visual analogue scale; miRNA: microRNA.

^a^Sample used only in miRNA screening.

^b^Sample used only in miRNA validation (all other samples were used for both miRNA screening and validation).

### miRNA expression analysis

**Figure 5.**
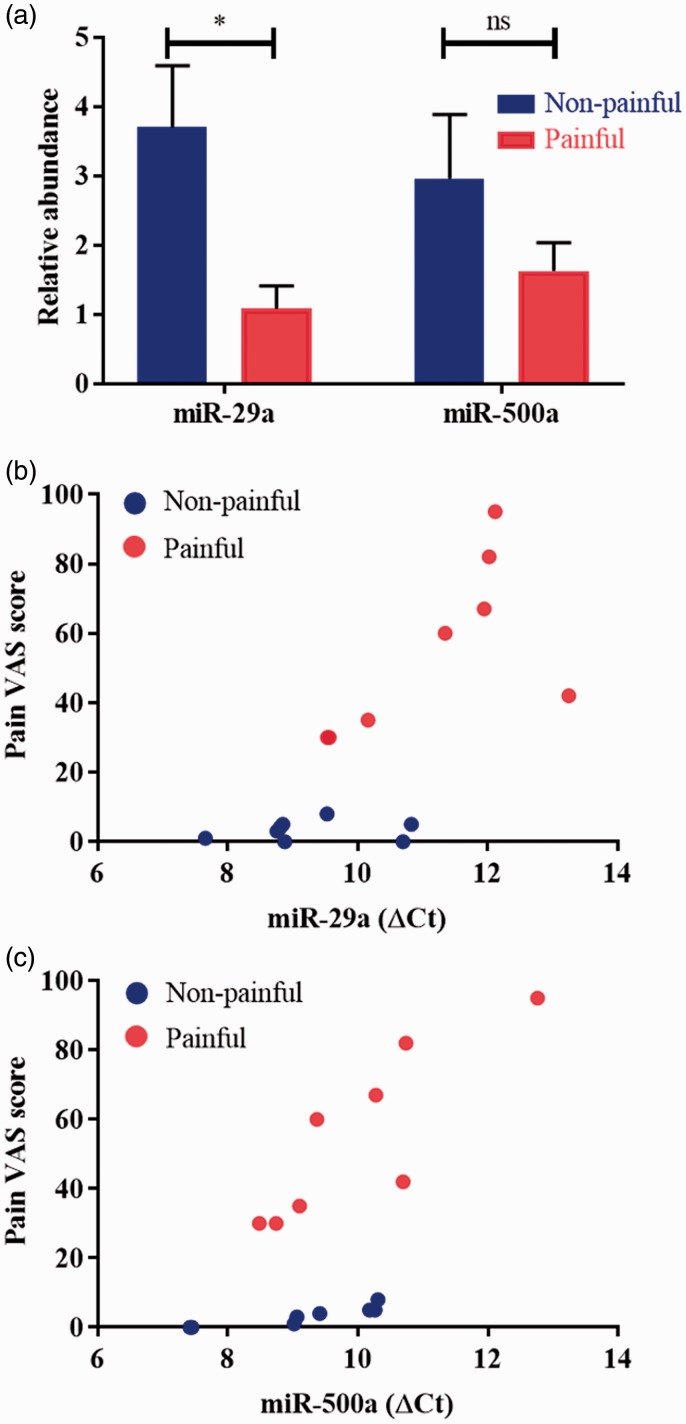
miRNA validation and correlation scatter plots. (a) miR-29a was confirmed to be statistically significantly differentially expressed (downregulated) in the painful group versus the non-painful group (*p = 0.02; unpaired *t* test). miR-500a was relatively downregulated in the painful group, but no statistical differences were found (ns; unpaired *t* test). (b and c) Pearson’s correlations showed a moderate inverse correlation between the expression of miR-29a and miR-500a, and the pain VAS scores (*r* = 0.7544, *p* = 0.0007 and *r* = 0.6515, *p* = 0.0063, respectively). In (b) and (c), miRNA values are given in terms of normalised ΔC_t_ values, where the higher the Ct value, the lower the expression of the miRNA. ns = not significant; VAS: visual analogue scale.

We identified two miRNAs, hsa-miR-29a and hsa-miR-500a, that were differentially expressed in painful and non-painful human lingual nerve neuromas in the TLDA screening analysis (Table 3). qPCR validation was conducted for these two miRNAs: miR-29a was found to be differentially downregulated in the painful group compared with the non-painful group (*t* = 2.76, *p* = 0.02; unpaired *t* test), while miR-500a was not statistically significantly different between the groups (Figure 5(a)). However, a significant positive correlation was found between delta C_t_ and the pain VAS score for both miRNA-29a (*r* = 0.75, *p* = 0.0007) and miRNA-500a (*r* = 0.65, *p* = 0.006) (Figure 5(b)), reflective of a negative correlation between abundance of these miRNA and pain. No correlation between tingling or discomfort and miRNA expression was observed.

**Table 3.** miRNA differentially expressed in human lingual nerve neuromas.

miRNA	ΔΔC_t_	*p* value
hsa-miR-29a	6.22	0.001
hsa-miR-500a	5.70	0.020

Human miRNA TLDA samples were analysed using the unpaired *t* test.

miRNA: microRNA.

### miRNA target prediction and gene enrichment analysis

**Figure 6.**
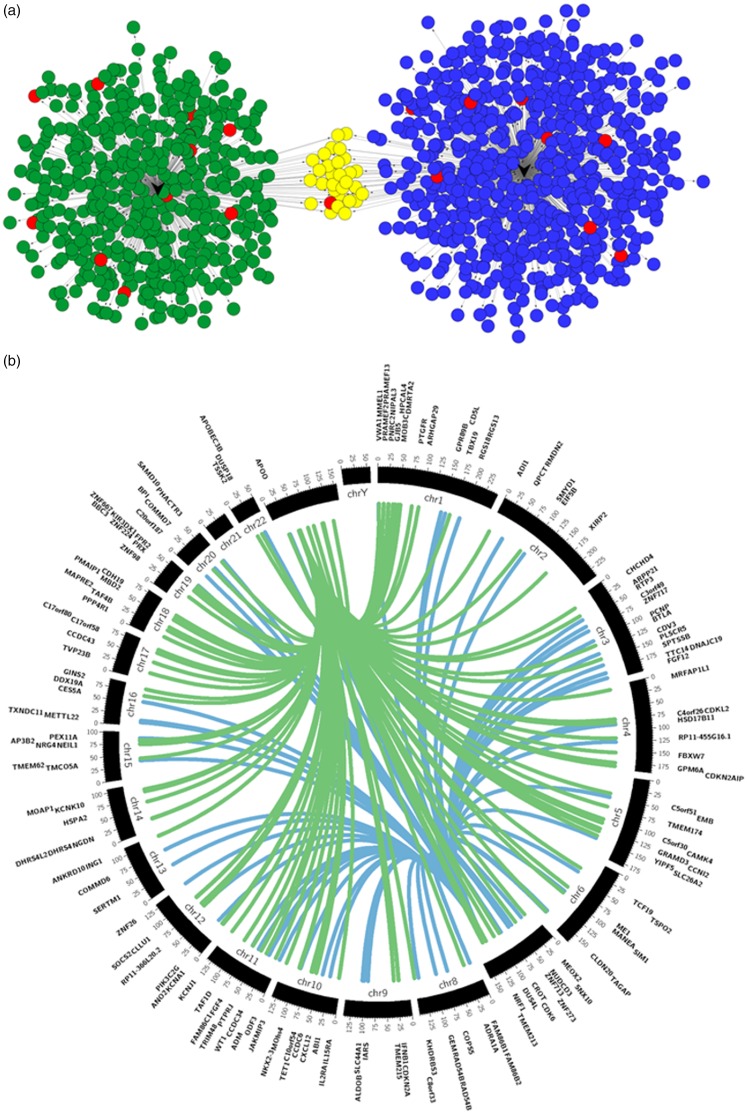
Human miRNAs of interest and respective putative target genes. (a) CyTargetLinker analysis and network visualisation of differentially expressed human neuromas miRNAs and their predicted target genes. Network enrichment genes for hsa-miR-29a and hsa-miR-500a were identified using MicroCosm, TargetScanHuman and miRTarBase. Nodes coloured in blue are targets of hsa-miR-29a, and nodes coloured in green are targets of hsa-miR-500a. The nodes coloured in yellow are targets of both hsa-miR-29a and hsa-miR-500a, while nodes in red are genes known to have a role in pain. This network was constructed and visualised using CyTargetLinker v3.0.1 and Cytoscape v3.4.0. (b) Circos v0.67 plot of hsa-miR-29a (blue) and hsa-miR-500a (green) and regulatory target genes. The middle track represents the human genome with each ideogram segment representing one chromosome. The outermost and inner tracks display the gene name and spatial location of each gene targeted by both human miRNAs.

Similarly to the process performed for the rat miRNAs, we also used the same publicly available databases to look for predicted target genes of the identified human miRNAs; both hsa-miR-29a and hsa-miR-500a were further analysed, given the correlation results. For visualisation purposes, the miRNA-target gene networks were again created with the CyTargetLinker app from Cytoscape, as represented in Figure 6(a). The gene targets chosen for further analysis were ranked by the cumulative weighted context++ scores of TargetScan (cut-off –0.4) resulting in 67 predicted targets for hsa-miR-29a and 115 for hsa-miR-500a. The predicted target genes are located across different chromosomes in the human genome (see Figure 6(b)), but both miRNAs share a number of common target genes. hsa-miR-500 is located on chromosome X, and hsa-miR-29a is located on chromosome 7 of the human genome.

Enrichment and pathway analysis were carried out using MetaCore™ process networks, pathway maps, GO molecular functions and GO biological processes for the human miRNA target genes identified. Examples of genes potentially targeted by the miRNAs and enriched for these pathways are listed in Table S2. Here, we report the most relevant pathways in the context of pain, nerve injury and inflammation. MetaCore process networks identified three main pathways: inflammation (Jak-STAT), potassium transport and chemotaxis. MetaCore pathway maps included IL-16 signalling pathway, IL-15 signalling via JAK-STAT cascade and impaired lipoxin A4 signalling. The enrichment analysis for GO biological process included regulation of transmembrane transport, G protein-activated inward rectifier potassium channel activity and macrophage apoptotic process; and negative regulation of leukocyte activation, leukocyte cell–cell adhesion, cell activation, cell growth and cell proliferation. GO molecular function identified several clusters associated with inflammation and ion and membrane transport including: IL-2 receptor activity and binding; cytokine receptor activity; ion channel activity; voltage-gated ion channel activity; transmembrane, substrate-specific and passive transmembrane transporter activity.

## Discussion

### Major findings

The following were the main findings of this study:

1. rno-miR-667 was increased in the lingual nerve of injured rats (CCI group) compared with sham control rats.
2. The abundance of rno-miR-138 and rno-miR-667 was found to be statistically significantly correlated (negatively and positively, respectively) with the time spent drinking on day 3 post-injury. A reduction in time spent drinking may indicate hypersensitivity to stimulation of the tongue (or other intraoral regions); thus, these data suggest that increased levels of rno-miR-138 and decreased levels of rno-miR-667 may be associated with presence of nerve injury-induced hypersensitivity.
3. hsa-miR-29a expression was reduced in lingual nerve neuromas of patients with higher pain VAS scores (painful group), compared with patients with lower pain VAS scores (non-painful group).
4. A statistically significant negative correlation was observed between the expression of both hsa-miR-29a and hsa-miR-500a, and the pain VAS score, indicating that reduced levels of both these miRNAs are associated with the presence of pain.
5. Target and gene enrichment analysis predicted that all the above miRNAs may target genes involved in inflammatory and pain-related pathways.

### miRNAs and hyperalgesic states

A range of previous studies have linked expression of miR-138, miR-667, miR-29a and miR-500 with changes related to nerve injury, other hyperalgesic states and neuroplasticity. It is difficult to directly compare these findings with those of the present study as there are differences in the models used, the time course and sites of assessment of miRNA expression, but these earlier studies do provide further evidence that the miRNAs identified in the present study may play a significant role in responses to nerve injury, hyperalgesia and neuroplasticity. The findings from these studies are summarised below.

#### miR-138

Work by Strickland et al.^46^ reports a downregulation in miR-138 in the rodent DRG seven days following sciatic nerve transection. miR-138 has also been linked more broadly with neuroplasticity. It is known to be highly expressed in brain synapses and has been shown to target the depalmitoylation enzyme acyl-protein thioesterase 1, which has been linked to the regulation of dendritic spine size in rat hippocampal neurons.^47^

#### miR-667

Previous studies also implicate altered miR-667 expression in the development of hyperalgesia. Lentiviral injections of this miRNA increase hypersensitivity in the mouse formalin model,^48^ a model of inflammatory pain. This is thought to be linked to the ability of miR-667 to reduce levels of catechol-O-methyltransferase (*Comt*) mRNA in the brain.^48^ COMT degrades catecholamines such as dopamine and epinephrine, and low levels of this enzyme have been associated with hyperalgesic states.^49^

#### miR-29a

Rodent studies report that miR-29a is decreased in spinal cord dorsal horn seven days after injury (CCI) to rat sciatic nerve.^50^ This miRNA has also been shown to be downregulated in the ipsilateral trigeminal ganglion 4 h after administration of CFA in a model of inflammatory muscle pain.^51^ miR-29a has also been associated with inflammatory pain conditions in man. It has been found in blood microvesicles, small bowel and colon tissues of irritable bowel syndrome patients. Its presence has been associated with increased intestinal permeability (linked with chronic visceral pain); inhibition of miR-29a expression in vitro restored intestinal permeability to normal levels.^52^

#### miR-500

It has been reported that miR-500 is downregulated in rat DRG after sciatic nerve resection at 4, 7 and 14 days^53^ and in the spinal cord dorsal horn neurons 7 days after CCI of sciatic nerve.^50^ How this relates to neuropathic pain is not yet clear, but there is some evidence that miR-500 downregulates the neurokinin-1 (substance P) receptor.^54^ Activation of this receptor by substance P produces mechanical hyperalgesia and allodynia,^55^ and these molecules have been implicated in neuropathic pain. Studies in our and others’ laboratories have shown that substance P accumulates at sites of nerve injury, including trigeminal nerve injury^27,28^ and application of substance P at these sites increases spontaneous activity.^26^ In another rodent study, miR-500 was shown to be increased after administration of paclitaxel or L5 ventral root transection and modulated the downregulation of glutamic acid decarboxylase 67 (GAD67),^56^ an enzyme that regulates the function of GABAergic synapses in spinal cord dorsal horn neurons. In the same study, miR-500 knockout or the use of an antagomir led to functional repair of GABAergic synapses and alleviated pain behaviour in rats, suggesting that miR-500 negatively contributes to GABAergic synaptic function through the regulation of GAD67. Other rodent studies have shown that miR-500 is expressed in the CNS, and it is thought to have a major role in CNS development.^57^

Studies in man also indicate a link between miR-500 and pain symptoms. Work conducted using human whole blood samples collected from patients with complex regional pain syndrome (CRPS) demonstrated that, even though miR-500 was not significantly altered between CRPS patients and controls, the levels of this miRNA were negatively correlated with the symptoms of hypoaesthesia.^58^ The miR-500 gene is located on chromosome X, and it is of interest that the majority of patients in the present study were female (Table 2). Recent evidence by Linnstaedt et al.^59^ shows miRNAs enriched for chromosome X in patients with persistent musculoskeletal pain, suggesting that miRNAs can play a role in sex-specific pain differences. There have also been some studies associating miRNAs in the X chromosome with potential sex-specific immune responses,^60^ and it has been shown that in several mammalian species, the Y chromosome has no miRNA genes. Further studies are required to elucidate the potential role of miRNAs in general, and miR-500a in particular, in sex-specific pain differences.

### Predictive target analysis

#### Potential cell types expressing miRNAs and predicted targets

The enrichment analysis of both rat and human miRNAs identified a wide range of predictive target genes involved in a range of processes including inflammation, chemotaxis, signal transduction and ion transmembrane transport. These processes are all influenced by tissue or nerve injury and can directly activate and/or sensitise nociceptors facilitating the transmission of nociceptive input^61^; they are thus potentially relevant to the development of neuropathic pain. The injured rat lingual nerves and human neuroma samples contain a range of structures. These include nerve axons, Schwann cells, fibroblasts, macrophages and other leukocytes.^37,62^ Further studies are required to identify which cell types are expressing the miRNAs shown to correlate with behavioural change and VAS scores for pain in the current study, but altered miRNA expression in any of the cell types listed above, or altered miRNA expression as a consequence of any change in the cell types present in the tissue (e.g., due to immune cell infiltration), has potential to impact on the development of pain. The neuronal somas and nuclei are not present in the rat and human nerve tissues used in this study; however, there is clear evidence that both neuronal miRNAs and mRNAs are present in neuronal axons.^63,64^ Of particular relevance to the findings from the present study, miR-138 and miR-29a are both reported to be abundantly expressed in sensory axons.^65^

#### Predictive targets of rodent miRNAs

Genes related to inflammation and chemotaxis that are thought to play key roles in nociceptive processing and neuropathic pain and are predictively targeted by rno-miR-138 and rno-miR-667 include the c-c motif chemokine ligand 3 (*Ccl3*, predictively targeted by rno-miR-138 with a 8mer canonical binding) and the c-x-c motif chemokine receptor 1 (*Cxcr1*, also known as il8ra, predictively targeted by rno-miR-667 with two possible canonical bindings: 7mer-A1 and 8mer). *Ccl3* is a cytokine gene located on chromosome 10 of the rat genome; it encodes CCL3, a protein that binds to several chemokine receptors, including chemokine binding protein 2 and chemokine (C-C motif) receptor 5. *Cxcr1* encodes CXCR1, which is a receptor for IL-8.

Genes related to ion transport potentially targeted by rno-miR-138 that have been linked with pain states include the transient receptor potential cation channel, subfamily C, member 1 (*Trpc1*) (8mer canonical binding) located on chromosome 8 in the rat genome. This is a nonspecific cation channel that contributes to nociceptor sensitisation.^66^ The enrichment analysis of rat miRNAs also identified predictive target genes involved in the Wnt signalling pathway. This pathway is known to participate in the regulation of neuron repulsion during axonal guidance and thus may be of relevance in axon regeneration following injury.^67^

#### Predictive targets of human miRNAs

Predictive target gene enrichment analysis of differentially expressed human miRNAs indicates that potassium voltage-gated channels are potentially targeted by hsa-miR-500a; thus, this miRNA may contribute to the regulation of several potassium channels. Many studies have demonstrated the role of potassium channels in diseases including pain.^68,69^ Genes predicted to be targeted by hsa-miR-500a include *Kcna1*, a gene located on chromosome 12 in the human genome that encodes for the potassium voltage-gated channel subfamily A member 1 (in addition to 8mer and 7mer-m8, a poorly conserved 6mer binding site is also predicted). *Kcnj1* (located in human genome chromosome 11) codes for an inwardly rectifying potassium channel (subfamily J) and is also predicted to be targeted by hsa-miR-500a (7mer-m8 canonical binding).

hsa-miR-29a was predicted to target genes that regulate inflammatory and immune mechanisms, mainly genes that encode G protein-coupled receptors (GPCRs) and IL receptors. For instance, the gene *FPR2*, located on chromosome 19, is a predicted target for this miRNA (an 8mer canonical binding). FPR2/ALX (a GPCR) is the related protein, and previous studies showed that activation of FPR2/ALX stopped the recruitment of neutrophils, facilitated resolution of inflammation and inhibited the release of cytokines and chemokines.^70,71^ Other studies suggest a role for FPR2/ALX in inhibiting pain processing.^72–74^ This receptor is classified as a multirecognition receptor as it binds to multiple molecules including lipoxin A4, resolvin D1, peptides and proteins (annexin and serum amyloid A), showing a ligand-specific effect.^75,76^ FPR2/ALX has been reported to be regulated by, at least, one other miRNA, the miR-181b, in human macrophages.^77^

### Methodological considerations

The sample sizes used in this study were relatively small, and this may explain the slight variation between the initial TLDA screening and the validation studies. However, despite the small sample sizes, clear correlations were shown between miRNA expression levels and drinking behaviour in rats, or VAS scores in patients. It should also be noted that the human neuromas had been fixed and stored at –80°C for periods of several months before miRNA extraction and analysis. However, several studies have demonstrated that miRNA is more stable than mRNA in fixed samples.^78–81^ The bioinformatics analysis was based on predicted targets rather than experimentally validated assays. However, the TargetScan algorithm used to predict targets in this study has been shown to have high concordance with experimental validation^82^ and has the advantage of giving genome-wide insight.

Different miRNAs were identified in the rat and in the human lingual nerve samples used; however, the miRNAs identified have predicted target genes involved in similar pathways. One possible explanation is the fact that rat lingual nerve tissues were collected and analysed in a specific time point (three days following injury), and human lingual neuroma tissues had variable time points between injury and tissue analysis (ranging from 7 to 36 months). miRNA expression is a dynamic process, and there is evidence that miRNAs can change in expression over time in the same tissue and pain condition.^9^ Furthermore, literature has suggested that the development and the maintenance of chronic pain is controlled by differing molecular mechanisms,^83,84^ denoting that different miRNAs may be involved at different stages.

## Summary

In summary, this study has demonstrated that specific miRNAs are differentially expressed in injured lingual nerves and that there are highly significant correlations between abundance of specific miRNAs, altered behaviour and pain VAS scores. To our knowledge, this is the first demonstration of correlations between human miRNA levels and VAS scores for neuropathic pain. Predictive analyses suggested that these miRNAs may interact with target genes that participate in inflammatory and signal transduction pathways and axon regrowth and thus may contribute to the molecular changes that occur after a peripheral nerve injury. Further functional studies are planned to evaluate possible physiological effects of these miRNAs in pain mechanisms.

## Supplemental Material

Supplemental material for Correlation of miRNA expression with intensity of neuropathic pain in man Click here for additional data file. Supplemental Material for Correlation of miRNA expression with intensity of neuropathic pain in man by Diana Tavares-Ferreira, Nathan Lawless, Emma V Bird, Simon Atkins, David Collier, Emanuele Sher, Karim Malki, Daniel W Lambert and Fiona M Boissonade in Molecular Pain

## Author Note

Current address for Diana Tavares-Ferreira is School of Behavioral and Brain Sciences, University of Texas at Dallas, Richardson, TX, USA.

Current address for Nathan Lawless is Neurological Diseases, Boehringer Ingelheim, Biberach, Germany.

Current address for Karim Malki is Institute of Psychiatry, Psychology & Neuroscience, King's College London, UK.
