# Supplementary material for: Correlation of miRNA expression with intensity of neuropathic pain in man
Source: Mol Pain. 2019 Jul 10;15:1744806919860323. doi: 10.1177/1744806919860323 (PMC6620726; doi:10.1177/1744806919860323)
Supplement: Supplemental material for Correlation of miRNA expression with intensity of neuropathic pain in man [file Supplemental_Material.pdf]

SUPPLEMENTS

Table S1. List of pathways and target genes enriched in rat lingual nerve.

**GO molecular function**	**Examples of target genes**	**p-value**
cation transmembrane transporter activity	Creatine transporter 1 (SLC6A8), Kv1.2, NPAL2, SLC35A4, ATP6V1G1, UQCRFS1, NRAMP1, TMEM38A, SLC39A11, GluR5, Kv8.1, Kir3.1, SLC39A9, TRPC1, CACNA1S, KCNJ14	3.84E-04
substrate-specific transmembrane transporter activity	Creatine transporter 1 (SLC6A8), Kv1.2, NPAL2, SLC35A4, ATP6V1G1, UQCRFS1, NRAMP1, TMEM38A, SLC39A11, SLC5A8, MIP26, GluR5, Kv8.1, GluR2, Kir3.1, SLC39A9, TRPC1, CACNA1S, SLC26A11, KCNJ14, CLCA4	4.103E-04
ion transmembrane transporter activity	Creatine transporter 1 (SLC6A8), Kv1.2, NPAL2, SLC35A4, ATP6V1G1, UQCRFS1, NRAMP1, TMEM38A, SLC39A11, GluR5, Kv8.1, GluR2, Kir3.1, SLC39A9, TRPC1, CACNA1S, SLC26A11, KCNJ14, CLCA4	4.347E-04
inorganic cation transmembrane transporter activity	Creatine transporter 1 (SLC6A8), Kv1.2, NPAL2, ATP6V1G1, UQCRFS1, NRAMP1, TMEM38A, SLC39A11, Kv8.1, Kir3.1, SLC39A9, TRPC1, CACNA1S, KCNJ14	5.547E-04
substrate-specific transporter activity	Creatine transporter 1 (SLC6A8), Kv1.2, NPAL2, SLC35A4, ATP6V1G1, UQCRFS1, NRAMP1, TMEM38A, SLC39A11, SLC5A8, ICAP-1, MIP26, MOG1, GluR5, Kv8.1, GluR2, Kir3.1, SLC39A9, TRPC1, CACNA1S, SLC26A11, KCNJ14, CLCA4	6.416E-04
transporter activity	Creatine transporter 1 (SLC6A8), Kv1.2, NPAL2, SLC35A4, ATP6V1G1, UQCRFS1, RENT3B, NRAMP1, TMEM38A, SLC39A11, SLC5A8, PC-TP, ICAP-1, MIP26, MOG1, GluR5, Kv8.1, NGAL, GluR2, Kir3.1, SLC39A9, TRPC1, CACNA1S, SLC26A11, KCNJ14, CLCA4	6.728E-04
voltage-gated cation channel activity	Kv1.2, GluR5, Kv8.1, Kir3.1, CACNA1S, KCNJ14	1.983E-03
potassium channel activity	Kv1.2, TMEM38A, Kv8.1, Kir3.1, KCNJ14	5.423E-03

ion channel binding	Syntaxin 1A, 14-3-3 theta, MOG1, Homer 1, TRPC1	6.18E-03
**GO biological process**	**Examples of target genes**	**p-value**
ion transport	Creatine transporter 1 (SLC6A8), Kv1.2, NPAL2, K(+) channel, subfamily J, SLC35A4, CCL3L1, ATP6V1G1, UQCRFS1, Galpha(i)-specific peptide GPCRs, NRAMP1, Syntaxin 1A, TMEM38A, SLC39A11, ATP6V1G, SLC5A8, Ionotropic glutamate receptor, MIP26, GluR5, CYB5R2, Kv8.1, NGAL, Galpha(q)-specific peptide GPCRs, RXR, SLC27A3, GluR2, Kir3.1, SLC39A9, TRPC1, Kainate receptor, IHPK2, CACNA1S, Galpha(q)-specific Class A Orphan/other GPCRs, SLC26A11, KCNJ14, 14-3-3, CLCA4, Dynamin	4.97E-08
regulation of transmembrane transport	Kv1.2, K(+) channel, subfamily J, Sts-1, Galpha(i)-specific peptide GPCRs, 14-3-3 theta, G-protein beta, Ionotropic glutamate receptor, MOG1, GluR5, Kv8.1, Galpha(q)-specific peptide GPCRs, Homer 1, DJ-1, Kir3.1, TRPC1, Kainate receptor, CACNA1S, KCNJ14, 14-3-3, Dynamin	2.038E-07
regulation of ion transmembrane transport	Kv1.2, K(+) channel, subfamily J, Sts-1, Galpha(i)-specific peptide GPCRs, 14-3-3 theta, G-protein beta, Ionotropic glutamate receptor, MOG1, GluR5, Kv8.1, Galpha(q)-specific peptide GPCRs, Homer 1, Kir3.1, TRPC1, Kainate receptor, CACNA1S, KCNJ14, 14-3-3, Dynamin	5.704E-07
regulation of ion transport	Neurokinin-2 receptor, Kv1.2, K(+) channel, subfamily J, CCL3L1, Sts-1, Galpha(i)-specific peptide GPCRs, 14-3-3 theta, G-protein beta, Ionotropic glutamate receptor, MOG1, GluR5, Kv8.1, Galpha(q)-specific peptide GPCRs, Homer 1, DJ-1, Kir3.1, TRPC1, Kainate receptor, CACNA1S, Galpha(q)-specific Class A Orphan/other GPCRs, KCNJ14, 14-3-3, Dynamin	1.512E-06
cellular response to interleukin-8, interleukin-8-mediated signaling pathway	Galpha(i)-specific peptide GPCRs, Galpha(q)-specific peptide GPCRs, IL8RA	2.83E-06

intracellular signal transduction	PAK3, RGS7, CRK, CCL3L1, Galpha(i)-specific peptide GPCRs, NRAMP1, RASAL3, VEGFR-2, HB-EGF, 14-3-3 theta, InPP5A, G-protein beta, CNIL, c-Rel (NF-kB subunit), MyD88, ICAP-1, p63, Miro-1, Rap1GDS1, RAP-2B, PLD2, SPEC1, CSF2RB, ZFP91, RET, Galpha(q)-specific peptide GPCRs, E2F7, PKA-reg type II (cAMP-dependent), DHC24, DJ-1, C9orf86, BIG2, Pleckstrin, Galpha(q)-specific Class A Orphan/other GPCRs, ASB6, PP2C, ERK3, PRKAR2A, 14-3-3, PKA-reg (cAMP-dependent)	3.93E-06
T cell chemotaxis	CCL3L1, Galpha(i)-specific peptide GPCRs, CXCL16, Galpha(q)-specific peptide GPCRs	4.23E-06
MetaCore pathways		
Cell adhesion_Chemokines and adhesion	CRK, VEGFR-2, IL8RA, Collagen IV	5.60E-03
**MetaCore networks**	**Examples of target genes**	**p-value**
Neurophysiological process_Transmission of nerve impulse	Galpha(i)-specific peptide GPCRs, Ionotropic glutamate receptor, GluR5, GluR2, Kainate receptor, PRKAR2A, PKA-reg (cAMP-dependent), GABARAPL1	3.59E-03
Signal transduction_WNT signaling	HB-EGF, HNF3-beta, p63, PKA-reg type II (cAMP-dependent), ERK3, PRKAR2A, PKA-reg (cAMP-dependent)	4.97E-03
Cell adhesion_Leucocyte chemotaxis	CCL3L1, Galpha(i)-specific peptide GPCRs, CXCL16, ICAP-1, PLD2, Galpha(q)-specific peptide GPCRs, IL8RA	1.06E-02
Chemotaxis	CCL3L1, Galpha(i)-specific peptide GPCRs, CXCL16, Galpha(q)-specific peptide GPCRs, IL8RA	2.34E-02
Transport_Calcium transport	Reticulocalbin 1, Ionotropic glutamate receptor, Homer 1, TRPC1, CACNA1S	7.829E-02
Inflammation_Neutrophil activation	Galpha(i)-specific peptide GPCRs, Syntaxin 1A, PLD2, IL8RA, PAK2	1.13E-01
Signal transduction_Neuropeptide signaling pathways	Neurokinin-2 receptor, Galpha(i)-specific peptide GPCRs, Galpha(q)-specific peptide GPCRs, PKA-reg (cAMP-dependent)	1.13E-01

MetaCore diseases biomarkers	Examples of target genes	p-value
Mouth Diseases	Neurokinin-2 receptor, Ro60, DTX4, FAM53C, K(+) channel, subfamily J, Syndecan-1, HSP47,FAM133B, NRAMP1, N-myristoyltransferase, CXCL16, KIAA1754L, PARD6G, VEGFR-2, G-protein beta, c-Rel (NF-kB subunit), COL4A1, GLE1, MyD88, ARPP- 21, ATP6V1G, NMT2, Ionotropic glutamate receptor, DAN, p63, PPTC7, Miro-1, CDH1, IL20RB, CSF2RB, ZFP91, Kv8.1, TRMT12, E2F7, PKA-reg type II (cAMP-dependent), RXR, GDF9, CYP19, PARD6, LRRC31, OKL38, IL8RA, TEX2, Galpha(t)-specific GPCRs, FRMPD1, CACNA1S, GPCRs, RXRG, PP2C, Collagen IV, PRKAR2A, 14-3-3, FAM46D, INTS2, CLCA4, PKA-reg (cAMP-dependent), ARNT2, Desmoplakin, DPH4, Dynamin	1.51E-05
Epilepsy, Generalized	K(+) channel, subfamily J, Galpha(i)-specific peptide GPCRs, Ionotropic glutamate receptor, GluR5, Kir3.1, Kainate receptor	2.24E-04

Table S2. List of pathways and target genes enriched in human lingual nerve neuromas

GO molecular function	Examples of target genes	p-value
interleukin-2 receptor activity and interleukin-2 binding	IL-2R alpha chain, sIL2RA	3.46E-04
cytokine receptor activity	IL-2R alpha chain, IL-15RA, sIL2RA, sIL-15RA	7.34E-03
ion channel activity	SLC26A2, ANO2, Kv1.1, CLIC4, KCNK10, GPM6A, SAP102, Kir1.1, GPR89B, GPR89A	2.97E-03
substrate-specific transmembrane transporter activity	SLC26A2, SLC44A1, SLC35E3, ANO2, Kv1.1, GLUT3, CLIC4, Embigin, KCNK10, GPM6A, SLC2A14, SAP102, C3orf55, Kir1.1, GPR89B, GPR89A, NPAL3	2.695E-03
gated channel activity	ANO2, Kv1.1, CLIC4, KCNK10, SAP102, Kir1.1, GPR89B, GPR89A	5.36E-03
voltage-gated channel activity	Kv1.1, CLIC4, KCNK10, Kir1.1, GPR89B, GPR89A	5.52E-03
voltage-gated ion channel activity	Kv1.1, CLIC4, KCNK10, Kir1.1, GPR89B, GPR89A	5.52E-03
channel activity	SLC26A2, ANO2, Kv1.1, CLIC4, KCNK10, GPM6A, SAP102, Kir1.1, GPR89B, GPR89A	6.42E-03
transmembrane transporter activity	SLC26A2, SLC44A1, SLC35E3, ANO2, Kv1.1, GLUT3, CLIC4, Embigin, KCNK10, GPM6A, SLC2A14, SAP102, C3orf55, Kir1.1, GPR89B, GPR89A, NPAL3	6.42E-03
substrate-specific transporter activity	SLC26A2, SLC44A1, SLC35E3, ANO2, Kv1.1, GLUT3, CLIC4, Embigin, PLSCR5, KCNK10, GPM6A, SLC2A14, SAP102, C3orf55, Kir1.1, GPR89B, GPR89A, NPAL3	6.50E-03
passive transmembrane transporter activity	SLC26A2, ANO2, Kv1.1, CLIC4, KCNK10, GPM6A, SAP102, Kir1.1, GPR89B, GPR89A	6.50E-03

GO biological process	Examples of target genes	p-value
regulation of ion transmembrane transport	K(+) channel, subfamily J, Kv1.1, CLIC4, KCNK10, FGF12, PI3K class II, Galpha(q)-specific prostanoid GPCRs, HSP70, Galpha(q)-specific peptide GPCRs, HSPA2, PDE, Kir1.1, GPR89B, GPR89A	3.212E-05
negative regulation of leukocyte cell-cell adhesion	IL-2R alpha chain, IFN-beta, BTLA, SDF-1, PDE, PP2135, p14ARF, p16INK4	3.865E-05
negative regulation of cell activation	IL-2R alpha chain, IFN-beta, BTLA, Galpha(q)-specific prostanoid GPCRs, Galpha(q)-specific peptide GPCRs, PDE, BPI, PP2135, p14ARF, p16INK4	4.23E-05
regulation of transmembrane transport	K(+) channel, subfamily J, Kv1.1, CLIC4, KCNK10, FGF12, PI3K class II, Galpha(q)-specific prostanoid GPCRs, HSP70, Galpha(q)-specific peptide GPCRs, HSPA2, PDE, Kir1.1, GPR89B, GPR89A	4.471E-05
regulation of G-protein activated inward rectifier potassium channel activity	K(+) channel, subfamily J, Kir1.1	4.851E-05
transmembrane transport	K(+) channel, subfamily J, SLC26A2, SLC44A1, SLC35E3, TAFs, ANO2, Kv1.1, SLC25A31, CHCHD4, GLUT3, CLIC4, Embigin, KCNK10, GPM6A, SLC2A14, HSP70, Galpha(q)-specific peptide GPCRs, PUMA, SAP102, C3orf55, PDE, Kir1.1, GPR89B, NPAL3	5.70E-05
negative regulation of leukocyte activation	IL-2R alpha chain, IFN-beta, BTLA, Galpha(q)-specific peptide GPCRs, PDE, BPI, PP2135, p14ARF, p16INK4	6.45E-05
negative regulation of cell growth	ING1, DEP-1, HSP70, Galpha(q)-specific peptide GPCRs, PUMA, CARF, WT1, p14ARF, p16INK4	6.648E-05
negative regulation of cell-cell adhesion	IL-2R alpha chain, IFN-beta, BTLA, Galpha(q)-specific prostanoid GPCRs, SDF-1, PDE, PP2135, p14ARF, p16INK4	6.65E-05

negative regulation of cell proliferation	IL-2R alpha chain, NOXA, TAFs, E3b1(ABI-1), FBXW7, ING1, DEP-1, Adrenomedullin, BTLA, HSP70, Galpha(q)-specific peptide GPCRs, Skp2/TrCP/FBXW, SAP102, PDE, PP2135, WT1, p14ARF, CDK6, p16INK4	8.76E-05
regulation of macrophage apoptotic process	Galpha(q)-specific peptide GPCRs, p14ARF, p16INK4	1.432E-04
**MetaCore pathways**	**Examples of target genes**	**p-value**
Immune response_T cell subsets: cell surface markers	IL-2R alpha chain, IL-15RA, BTLA	1.49E-03
Immune response_IL-16 signaling pathway	IL-2R alpha chain, Skp2/TrCP/FBXW, SDF-1	1.75E-03
Immune response_IL-15 signaling via JAK-STAT cascade	IL-15RA, sIL-15RA	4.08E-03
Impaired Lipoxin A4 signaling in CF	SOCS2, FPRL1	1.572E-02
Development_Lipoxin inhibitory action on PDGF, EGF and LTD4 signaling	SOCS2, FPRL1	1.071E-02
**MetaCore networks**	**Examples of target genes**	**p-value**
Inflammation_Jak-STAT Pathway	IL-15RA, SOCS2, IFN-beta, SDF-1	8.12E-02
Transport_Potassium transport	K(+) channel, subfamily J, Kv1.1, KCNK10, Kir1.1	9.15E-02
Chemotaxis	FPRL1, Galpha(q)-specific peptide GPCRs, SDF-1	1.20E-01
